# Proposed Extension to Professional Classes

**Published:** 1921-03-05

**Authors:** 


### DISTRICT NURSING IN LONDON.

### Proposed Extension to Professional Classes.

The importance and possibilities of the work of the Central Council for District Nursing in London are vividly brought out by a perusal of the sixth annual report for the year to December 31, 1920, and the report of the Executive Committee, which were presented to the annual meeting held in the Board Room of the Metropolitan Asylums Board on Monday, February 28. The Council's report deals first with the changed economic conditions of the country and the lack of accommodation for resident nurses, both of which causes contribute to the necessity to provide nurses for people who do not usually seek the services of district nurses. The Council has found that the shortage of district nurses continues, and its panel of emergency nurses has been amply taken advantage of during the year 1919-20 by various district nursing associations, which have absorbed the temporary services of thirty nurses for holidays and other special needs. The minimum salary of £50 which was formerly insisted on as a condition of a grant from the Council to an Association has been raised to £63. The necessary three years' general training is not considered sufficient for such varied and comprehensive work as that falling to the district nurse, who, it is pointed out, has a direct share in all the health work of her district, including infant welfare, tuberculosis, and the prevention as well as cure of disease.

### Further Support Needed.

The Council reports the receipt of £10,000 from the Joint Committee of the British Red Cross and the Order of St. John, £1,500 of which has been distributed to the Associations in respect of work done, and £1,000 for special capital expenditure. The remaining £7,500 has been invested. The one condition of the grant was that special consideration should be given to work done amongst ex-Service men. £2,300 has been received from the trustees of the London Parochial Charities, £500 from the London Insurance Committee, given with the hope that nursing services might be provided for insured persons all over the county of London, and grants from the Peabody and Guinness Trustees in recognition of services rendered by district nurses to residents in the buildings of these trusts. The Ministry of Health granted £375 in respect of the midwifery and maternity nursing carried out by certain associations. The Council has been enabled to make increased grants (they amounted altogether to £5,505), but, despite this, several of the associations find it necessary to curtail their work.

### Retirement of Miss Puxley.

At the meeting on February 28 Sir William Collins, M.D., K.C.V.O., was elected Chairman of the Council for the ensuing year, and occupied the chair. Sir Thomas Barlow was elected Vice-Chairman, and Miss Amy Hughes and Miss Irene Hett Honorary Secretaries. Great regret was expressed that Miss Zoe Puxley, Joint Honorary Secretary of the Council since its formation, desires to retire. Miss Puxley, in a happy speech, referred to the beginnings of the Council eight years ago, when, at the instance of Sir Arthur Downes, then Medical Inspector to the Local Government Board, Mr. John Burns, the then President, took steps to bring into co-ordination the various bodies and individuals responsible for district nursing in London. The Central Council for District Nursing was the result. Proposed by the Chairman of the Executive Committee (Mr. E. B. Turner, F.R.C.S.), and seconded by Miss Amy Hughes, the Council's thanks were voted to Miss Puxley for "her invaluable services as Honorary Secretary to this Council for the last seven years."

### Scheme for Visiting Nurses.

The motion that the report of the Executive Committee be received was proposed by Sir William Collins, and the various recommendations it contains were put to the meeting one by one. One representative of the British Red Cross Society and Order of St. John, and one of the College of Nursing are to be added to the Council. An important part of the Executive Committee's report is its memorandum on visiting nurses, which has been circulated in the nursing and medical papers to the District Nursing Associations and the Nurses' Co-operation. This was published in full in our issue of November 13 last, page 154. It outlines a scheme for the nursing care of three classes of patients: (1) those who are without available accommodation for a private nurse, even if able to pay full fees; (2) patients of the professional classes unable to afford the fees of a private nurse; and (3) tradespeople and others not included in the work of the district nurse, but urgently in need of some such assistance. It is recognised that services rendered by District Nursing Associations should be limited to those who cannot pay the full fees necessary to put the scheme on to a profit-earning basis. But a large number of the cases fall below this level, and the Central Council considers that a scheme might be usefully drawn up for London districts. An organisation such as the Nurses' Co-operation is suggested for visiting nurses nursing patients in class (1); a suggested scale of charges is 3s. 6d. a visit, or 5s. for two daily visits, and a proportionate reduction for a week; at this rate the weekly earnings of a visiting nurse would be four to six guineas, 30s. of which it is estimated would be required for board and lodging when living as a member of a small district community; the earnings beyond £3 3s. could be reserved for insurance, sick fund, etc., any excess after the satisfaction of these claims being divided as a bonus. Cases in classes (2) and (3) would, it is suggested, be dealt with by District Nursing Associations at a somewhat lower rate, say 2s. 6d. or 3s. for a single visit. The Council hopes that action will be taken to meet the need by means of some such scheme, and the matter is now being considered by several of the Associations.

### Special District Training.

The Executive Committee considers that the attention of the nurse-training-schools should be drawn to the large amount of additional instruction needed to equip a nurse for district work, and its recommendation that this view should be communicated to the General Nursing Council for consideration in connection with the training of nurses was adopted by the Central Council. In dealing with the recommendations that a condition of the Council's grant should be that the minimum salary for a district nurse shall be £63 clear over and above a reasonable allowance for board and lodging, it was decided, on the motion of Mrs. Handel Booth, that, in addition to board and lodging, an allowance for uniform and laundry should be made. Mr. David Pennant, of the Queen Victoria Jubilee Nurses' Institute, speaking on the Executive Committee's recommendation to consider the desirability of an appeal to the public for funds in aid of district nursing in London, desired to add an instruction to the Committee to confer with the Jubilee Institute in regard to the question of contributions from approved Societies. These Societies, he pointed out, are directly concerned in the prevention of sickness which the timely ministrations of the nurse can often effect; they now have the necessary funds in the declared surplus under the valuation which is proceeding; and the present is the moment to consider such a proposal. This was confirmed later by Mrs. Handel Booth, a representative of the Insurance Committee for the County of London, who, speaking unofficially, expressed her personal belief that if some really complete scheme were offered to the Approved Societies it would be welcomed for consideration. She believes there is a strong feeling amongst the Approved Societies in favour of nursing benefit in the disposal of the surplus moneys. Mr. Pennant's addition to the recommendation was accepted.

On the presentation of the Finance Committee's report by its Vice-Chairman, Monsignor Carton de Wiart, Sir William Collins acknowledged the great help of the Ministry of Health in providing office accommodation and other facilities for the Council's work.

